# Is the Brain a Key Player in Glucose Regulation and Development of Type 2 Diabetes?

**DOI:** 10.3389/fphys.2019.00457

**Published:** 2019-04-26

**Authors:** Martin H. Lundqvist, Kristina Almby, Niclas Abrahamsson, Jan W. Eriksson

**Affiliations:** Department of Medical Sciences, Uppsala University, Uppsala, Sweden

**Keywords:** CNS, hypothalamus, glucose, regulation, fMRI, neuroimaging, neuroendocrine, autonomic nervous system

## Abstract

Ever since Claude Bernards discovery in the mid 19th-century that a lesion in the floor of the third ventricle in dogs led to altered systemic glucose levels, a role of the CNS in whole-body glucose regulation has been acknowledged. However, this finding was later overshadowed by the isolation of pancreatic hormones in the 20th century. Since then, the understanding of glucose homeostasis and pathology has primarily evolved around peripheral mechanism. Due to scientific advances over these last few decades, however, increasing attention has been given to the possibility of the brain as a key player in glucose regulation and the pathogenesis of metabolic disorders such as type 2 diabetes. Studies of animals have enabled detailed neuroanatomical mapping of CNS structures involved in glucose regulation and key neuronal circuits and intracellular pathways have been identified. Furthermore, the development of neuroimaging techniques has provided methods to measure changes of activity in specific CNS regions upon diverse metabolic challenges in humans. In this narrative review, we discuss the available evidence on the topic. We conclude that there is much evidence in favor of active CNS involvement in glucose homeostasis but the relative importance of central vs. peripheral mechanisms remains to be elucidated. An increased understanding of this field may lead to new CNS-focusing pharmacologic strategies in the treatment of type 2 diabetes.

## Introduction

The global prevalence of diabetes in adults – approximately 90% consisting of type 2 diabetes – was estimated to 6.4% in 2010 and is predicted to increase to 7.7% in 2030 (Nolan et al., 2011). The macro- and microvascular complications that are associated with diabetes lead to increased morbidity and mortality and the economic burden posed by management of diabetes and its complications is substantial (Ng et al., 2014; Norhammar et al., 2016). Type 2 diabetes typically evolves gradually. An initial phase of insulin resistance with maintained normoglycemia is followed by a transitional phase of impaired fasting glucose and/or impaired glucose tolerance until manifest diabetes is established. While the pancreatic beta cells can compensate for the insulin resistance by increasing insulin secretion at first, they eventually fail to do so as the disease progresses, frequently necessitating exogenously administered insulin in advanced stages. Since the discovery of the pancreatic hormones insulin and glucagon, the prevailing understanding of type 2 diabetes development has circled around processes in the periphery, particularly in the pancreas. Likewise, pharmacological targets in the treatment of type 2 diabetes have been largely limited to the peripheral domain. However, this “islet-centric” model has these last decades been challenged by mounting evidence in favor of a “brain-centric” model, according to which the brain is actively involved in systemic glucose regulation. Further advances in this area may change the way we look at metabolic disorders and may specifically result in new CNS-targeted strategies for the pharmacological management of type 2 diabetes. In this narrative review, we aim to present the current knowledge of the field. In the first section, we will provide a brief summary of findings from animal studies that have been extensively reviewed by other authors (Marty et al., 2007; Carey et al., 2013; Grayson et al., 2013; Mergenthaler et al., 2013; Roh et al., 2016; Tups et al., 2017; López-Gambero et al., 2019). This will be followed up by a more in-depth presentation of evidence from human studies where the implementation and advances of neuroimaging techniques has offered new and interesting insights.

## Evidence From Animal Studies

In 1854, Claude Bernard reported that a lesion in the floor of the fourth ventricle in dogs altered glucose levels, thereby presenting the first evidence of the brain’s role in glucose regulation (Bernard, 1855). In the 1960s two sets of neurons were identified in the CNS that responded to high and low values of glucose, respectively (Anand et al., 1964; Oomura et al., 1964, 1969, 1974). These neurons were subsequently termed glucose-excitatory (GE, responding to high levels of glucose) and glucose-inhibitory (GI, responding to low levels of glucose) (Routh et al., 2014). While present in the entire CNS, these neurons are especially numerous in several nuclei of the hypothalamus and the brainstem (López-Gambero et al., 2019).

**FIGURE 1:**
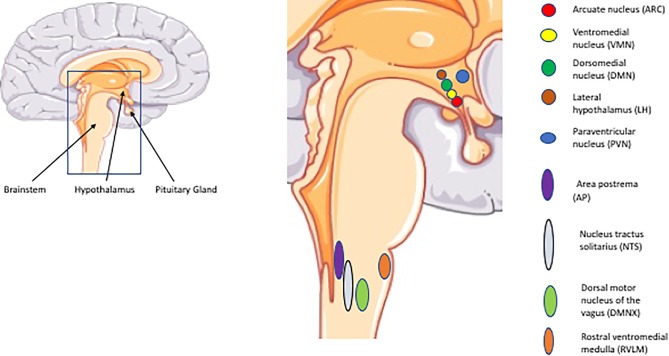
Important CNS regions and nuclei implicated in glucose regulation. The illustrations of anatomical structures were retrieved from http://smart.servier.com and have thereafter been assembled and processed.

The hypothalamus is located below the thalamus and above the pituitary gland and brain stem. It constitutes the floor of the third ventricle which contains cerebrospinal fluid (CSF). This anatomical position allows for access to nutrients and hormones. It consists of a network of interconnected nuclei among which the arcuate nucleus (ARC), ventromedial hypothalamus (VMH), dorsomedial nucleus (DMN), paraventricular nucleus (PVN), and the lateral hypothalamus (LH) are implicated in the regulation of glucose homeostasis. In the brainstem the nucleus of the solitary tract (NTS), area postrema (AP), dorsal motor nucleus of the vagus (DMNX) and the rostral ventrolateral medulla (RVLM) are key regions (López-Gambero et al., 2019). Glucose-excited neurons are primarily prevalent in the ARC, VMH, and PVN, whereas glucose-inhibited neurons are concentrated in the LH, AN, and PVN. In the brainstem, both of these types of neurons are represented in the areas mentioned above (NTS, AP, and DMNX) (Roh et al., 2016) (Figure 1).

Glucose injected into the third ventricle of rodents leads to a reduction of systemic glucose levels, principally by suppression of hepatic glucose production (HGP) (Lam et al., 2005a). The same has been demonstrated for long-chain fatty acids (Obici et al., 2002b; Lam et al., 2005b) and amino acids such as leucine (Su et al., 2012), proline (Arrieta-Cruz et al., 2013) and histidine (Kimura et al., 2013). Apart from being able to sense levels of circulating glucose, the brain also receives afferent nervous input via the brainstem related to glucose availability from taste buds in the oral cavity (Ahrén, 2000), from the intestinal mucosa (Ashley Blackshaw and Young, 2011) and from hepatoportal glucoreceptors (Delaere et al., 2010). This allows for rapid responses to ingested and circulating glucose such as observed in the cephalic insulin phase (Marty et al., 2007). Likewise, ingestion of lipids leads to release of CCK whereas amino acids stimulate release of CCK, GLP-1, and PYY. These gut hormones in turn activate vagal afferents projecting to the brainstem (Duca and Yue, 2014; Liu et al., 2016).

Not only neurons but also surrounding glial cells are believed to have an active role in the sensing of nutrients. It has been hypothesized that astrocytes may metabolize glucose and proline into lactate for subsequent translocation in the so-called astrocyte-neuron-lactate-shuttle to neurons to signal glucose depletion (Arrieta-Cruz et al., 2013). Likewise, oxidation of fatty acids may occur to a larger extent in astrocytes, generating ketones which after translocation to neurons may override the influence of glucose and fatty acid sensing (Bruce et al., 2017).

Similarly, central administration of hormones or targeting of their intracellular pathways in neurons have demonstrated that insulin (Obici et al., 2002a,c; Pocai et al., 2005), leptin (Schwartz et al., 1996; Kievit et al., 2006; Koch et al., 2010), glucagon (Mighiu et al., 2013; LaPierre et al., 2015), GLP-1 (Sandoval et al., 2008) and CCK (Zhu et al., 2012; Montgomery et al., 2013) reduces systemic glucose levels by CNS actions, whereas the effect of ghrelin seems to be the opposite (Scott et al., 2012; Wang et al., 2013; Stark et al., 2015; Lee et al., 2016). Perhaps most interesting is the potent CNS-mediated glucose lowering properties of the fibroblast growth factors FGF-1 (Scarlett et al., 2016) and FGF-19 (Fu et al., 2004; Morton et al., 2013; Marcelin et al., 2014). A single low dose of FGF-1 injected into the third ventricle induced sustained diabetic remission in mice with diet-induced obesity. The effect was rapid and sustained. Thus, fasting blood glucose concentration was reduced by 25% within 6 h, normalized after 7 days and remained in the normal range for the full 17 weeks studied without any observed hypoglycemia (Scarlett et al., 2016).

The transport of glucose across the blood–brain barrier (BBB) is primarily mediated by insulin-independent GLUT-1 and, particularly in conditions of CNS ischemia and glucose deprivation, by SGLT-1 (Patching, 2017). Interestingly though, insulin-mediated increase in CNS glucose uptake via upregulation of GLUT-1 in astrocytes has been described (García-Cáceres et al., 2016). Additionally, insulin-dependent GLUT4 is expressed in cells of the BBB, albeit at low levels (Patching, 2017). The existence of a more direct route, bypassing the BBB by fenestrated capillaries or via the CSF has been proposed and is supported by some work (Langlet et al., 2013; Routh et al., 2014). Fatty acids and amino acids have been demonstrated to cross the BBB (Smith, 2000; Rapoport et al., 2001; Smith and Nagura, 2001) by mechanisms that are not fully understood but may involve fatty acid transport proteins (FATPs) (Mitchell et al., 2011) and similar systems for amino acids (Smith, 2000). The levels of the large hormones insulin and leptin are considerably lower in the CFS than in the systemic circulation, suggesting the existence of saturable transporter proteins rather than simple diffusion (Banks et al., 1996; Gray and Barrett, 2018). While the exact mechanisms of CNS transport for these hormones remain elusive, hormone receptors seem to be involved (Banks et al., 1997; Bjørbaek et al., 1998; Boado et al., 1998; Hileman et al., 2002; Meijer et al., 2016). GLP-1 is synthesized within the CNS where it acts as transmitter (Katsurada et al., 2014; Katsurada and Yada, 2016), whereas the hypothesized intracerebral insulin synthesis is still a matter of some controversy (Gray and Barrett, 2018).

The intracellular mechanisms of nutrient and hormonal sensing in the CNS have been intensively investigated in animal studies. Glucose uptake into neurons occurs mainly via insulin-independent glucose transporter channels (GLUT1, 2, and 3) and this activates signaling pathways that differ for GE and GI neurons, involving activation of K_ATP_-channels in the former and the signaling substance AMPK in the latter. In GE neurons, metabolism-independent sensing mechanisms involving SGLT1 and 3 and the sweet receptor T1R2/3 are also implicated (López-Gambero et al., 2019). The receptors of insulin (Chen et al., 2017), leptin (Mercer et al., 1996), glucagon (Abraham and Lam, 2016), GLP-1 (Katsurada and Yada, 2016), CCK (Pathak et al., 2018), and ghrelin (Cowley et al., 2003; Sun et al., 2007) have been identified in various regions of the CNS. Insulin signaling in neurons is dependent on activation of K_ATP_-channels (Pocai et al., 2005) and is facilitated by activation of the IRS-PI3K- pathway (Niswender et al., 2003). Leptin also acts on this pathway by phosphorylation of IRS1 (Koch et al., 2010) in addition to the JAK2-STAT3 and WNT signaling pathways (Tups et al., 2017). Thus, leptin may sensitize neurons in the hypothalamus to the effects of insulin but may also exert central effects independent of insulin, as is supported by the fact that leptin can reverse hyperglycemia in mice with streptozotocin-induced type 1 diabetes (Yu et al., 2008).

There is much evidence that the central effects of insulin and leptin differ in obese compared to lean animals. Impairments in CNS transport, receptor expression and the downstream signaling cascades have been observed for both hormones in models of obesity (Van Heek et al., 1997; El-Haschimi et al., 2000; Kaiyala et al., 2000; Spanswick et al., 2000; Carvalheira et al., 2003; Asilmaz et al., 2004; Wilsey and Scarpace, 2004; Urayama and Banks, 2008). Hypothalamic inflammation has been proposed as a causative factor. Increased expression of inflammatory mediators such as TNF-alpha and increased endoplasmatic reticulum (ER) stress is a well-established feature of obesity (Hotamisligil et al., 1993; Ozcan et al., 2004). Cytokines and ER stress lead to activation of the intracellular JNK1 and IKKβ/NFkB pathways. This has been demonstrated to induce insulin resistance in peripheral tissues through serine phosphorylation of IRS-1. Obesity and over-nutrition is associated with hypothalamic inflammation, which may in turn result in insulin and leptin resistance via the mechanisms described above (Hosoi et al., 2008; Zhang et al., 2008; Milanski et al., 2009; Ozcan et al., 2009; Posey et al., 2009). Indeed, central inhibition of IKKβ has been observed to reduce food intake and increase insulin sensitivity (Zhang et al., 2008; Ozcan et al., 2009; Posey et al., 2009). Moreover, structural changes to the hypothalamus and evidence of increased inflammation have been observed well before the onset of obesity (Zhang et al., 2008; Thaler et al., 2012). Free fatty acids may also activate these pathways as well as activate macrophages by binding to toll-like receptors (TLRs) on their cell surface (Hotamisligil, 2006). High fat feeding, selective intake of saturated fatty acids and ICV infusion of palmitate have been associated with hypothalamic inflammation independently of excess caloric intake (Milanski et al., 2009; Posey et al., 2009). Finally, switching the diet to more unsaturated fats was observed to reduce this inflammation as well as hypothalamic and systemic insulin resistance (Cintra et al., 2012).

The role of inflammation in obesity is still disputed and may be multi-faceted, as is demonstrated by the positive effects of the cytokines IL-6 and IL-10 on energy and glucose metabolism, which seem to be partly mediated by central mechanisms (Wallenius et al., 2002; Benrick et al., 2009; Gotoh et al., 2012; Timper et al., 2017). The expression and circulating levels of these cytokines are increased in obese humans (Kern et al., 2001; Vozarova et al., 2001; Esposito et al., 2003). IL-6 administered to mice intracerebroventricularly was recently shown to induce reduced food intake and improved glucose metabolism, the latter mediated by enhanced hepatic insulin action. Moreover, this was more marked in obese, leptin-resistant mice than in lean ones (Timper et al., 2017). Thus, the increased levels of these cytokines may actually counteract rather than add to the other detrimental metabolic effects of obesity. Of further interest, IL-6 secretion by myocytes increases considerably after physical exercise and leads to subsequent increase in circulating levels of IL-10, secreted by immune cells (Steensberg et al., 2003; Pedersen and Febbraio, 2008). IL-6 and IL-10 have been shown to mediate positive metabolic effects of exercise through inhibition of IKKβ and ER stress in the hypothalamus (Ropelle et al., 2010).

**FIGURE 2:**
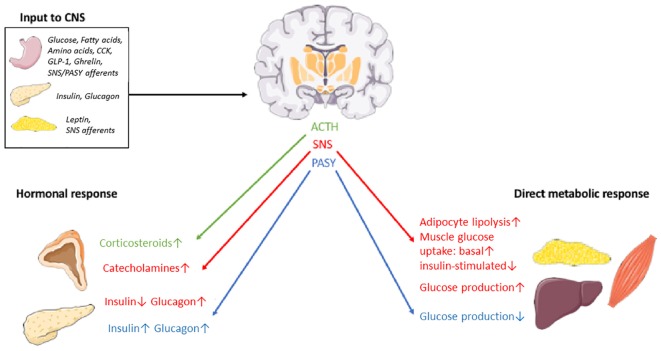
Schematic overview of some organs, nutrients, hormones and effector pathways involved in the postulated CNS-coordinated glucose regulation. SNS, sympathetic nervous system. PASY, parasympathetic nervous system. The illustrations of anatomical structures were retrieved from http://smart.servier.com and have thereafter been assembled and processed.

In order to exert its proposed influence on peripheral glucose metabolism, the CNS input from nutrients, hormones and other neurons has to result in an efferent response that is conveyed to peripheral target organs. The known routes for this response is the autonomic nervous system (ANS) – further divided into the sympathetic nervous system (SNS) and the parasympathetic nervous system (PASY) – and the hypothalamic-pituitary axis (Figure 2).

The DMNX of the brainstem contains the soma of preganglionic parasympathetic nerve fibers and can thus transmit PASY signals to target organs like the liver (Yi et al., 2010) (reducing hepatic glucose production and increasing glycogen synthesis) and the pancreas (Thorens, 2014) (increasing insulin secretion and glucagon secretion). Neurons in the RVLM connect with the SNS to increase hepatic glucose production (HGP) (Yi et al., 2010), decrease insulin secretion and increase glucagon secretion (Thorens, 2014), promote glucose uptake in skeletal muscles (Seoane-Collazo et al., 2015) and lipolysis in adipocytes (Bartness et al., 2005). Neurons in the PVN engage in the hypothalamic-pituitary-adrenal axis by secreting corticotrophin releasing hormone (CRH) in the bloodstream for downstream release of ACTH in the pituitary and ultimately secretion of glucocorticoids from the adrenal cortex. TRH, somatostatin, vasopressin and oxytocin is also synthesized in the PVN and may also contribute to long-term regulation of energy and glucose metabolism (Roh et al., 2016; López-Gambero et al., 2019). Finally, growth hormone (GH) release from the pituitary is stimulated by growth hormone-releasing hormone (GHRH) and inhibited by somatostatin, both released from the hypothalamus. The increase in GH levels observed during hypoglycemia seem to be dependent on alpha-adrenergic dependent somatostatin inhibition and GHRH stimulation (Lim and Khoo, 2000). GH raises glucose levels in the periphery by essentially counteracting insulin. This effect is, however, opposed by the insulin-like action of IGF-1, release of which from the liver is stimulated by GH (Clemmons, 2012). Nevertheless, type 2 diabetes is a distinct clinical consequence of GH excess in acromegaly (Hannon et al., 2017), attesting to a net hyperglycemic effect of the growth hormone axis. A subset of neurons in the ARC express GHRH and seem to be involved in the regulation of GH secretion in a complex interplay with POMC-, NPY- and somatostatin-expressing neurons (Steyn et al., 2016). Indeed, POMC and NPY/AgRP neurons are implicated in CNS-mediated glucose and energy regulation, acting in opposite directions (Tups et al., 2017). Thus, the hypothalamus-pituitary-growth hormone axis may provide an additional effector pathway from the brain to the periphery that influences systemic glucose homeostasis. By binding to the receptor GHSR on GHRH neurons, ghrelin may interfere with this pathway along with a direct effect on NPY/AgRP neurons (Steyn et al., 2016).

Evidence of CNS involvement in glucose homeostasis also comes from a study in which mice that moved from a warm to a cold environment had a coordinated reduction in both insulin secretion and insulin sensitivity, despite the absence of hypoglycemia. This was reversed within 4 h of returning to room temperature and within 30 min of administration of the alpha-adrenergic blocker phentolamine. According to the authors, the results imply a key role of the brain in rapidly coordinating insulin secretion and insulin sensitivity via the SNS (Morton et al., 2017). The control of body temperature is coordinated in the hypothalamus (Morrison, 2016) and crosstalk between this neuronal circuitry and those that are supposedly involved in glucose regulation would not be surprising.

## Evidence From Studies in Humans

### Clinical Observations in CNS and Metabolic Disorders

There is an intriguing epidemiological correlation between features of the metabolic syndrome and various diseases affecting the CNS of humans that gives indirect support to the brain’s involvement in peripheral glucose regulation. Depression is twice as common in patients with type 2 diabetes (Moulton et al., 2015). A correlation between depression and insulin resistance that was attenuated when adjusting for bodyweight and other confounders was reported in a meta-analysis from 2013 (Kan et al., 2013). In a longitudinal study, depression in middle-aged women was associated with increased insulin resistance and increased incidence of diabetes type 2, both of which were explained by increased central adiposity (Everson-Rose et al., 2004). Other longitudinal studies have highlighted an increased risk in patients with depression to develop the metabolic syndrome and/or its components (Marazziti et al., 2014).

An increased prevalence of the metabolic syndrome has also been reported repeatedly in studies of patients with psychotic illness including schizophrenia (Newcomer, 2007). While this may be partly due to the well-known side effects of antipsychotics, a meta-analysis from 2016 reported that first-episode psychosis was associated with increased insulin resistance and impaired glucose tolerance (Perry et al., 2016). Similar associations have been observed for anxiety and bipolar disorder (Czepielewski et al., 2013; Smith et al., 2013) while studies addressing the connection between work-related stress and development of diabetes type 2 have been inconsistent (Cosgrove et al., 2012; Krajnak, 2014). Patients with type 2 diabetes also have an increased risk of developing dementia, particularly vascular dementia and Alzheimer’s disease and it has been hypothesized that insulin resistance in the brain contributes to the development of Alzheimer’s disease (Li et al., 2015; Chatterjee and Mudher, 2018).

Naturally, epidemiological observations such as these do not prove causation, still leaving us with the question: does CNS diseases cause metabolic disorders or vice versa? In some cases, there is probably a bidirectional causality, e.g., for Alzheimer’s disease and type 2 diabetes (Shinohara and Sato, 2017). However, one plausible causal mechanism is activation of the stress system (primarily the ANS and the hypothalamic-pituitary-adrenal axis) in psychiatric and neurodegenerative diseases leading to metabolic consequences down the line. As outlined previously, these two systems constitute efferent pathways by which the brain may exert its putative influence on whole-body glucose homeostasis. Over-activation of either or both of these systems have been postulated to contribute to the development of metabolic disorders.

### Disturbances of the Autonomic Nervous System in CNS and Metabolic Disorders

As previously described, the ANS consists of the SNS and the PASY. It has a range of biologic effects including influencing glucose homeostasis in the periphery. Activation of the SNS raises circulating glucose levels by inhibiting insulin release from beta cells, stimulating release of glucagon from alpha cells, increasing release of epinephrine from the adrenal glands (augmenting and prolonging the effect of nervous activity) and increasing hepatic glucose production. In contrast, PASY activation lowers glucose levels by stimulating the secretion of insulin from beta cells and suppressing hepatic glucose production, while similarly increasing glucagon levels (Roh et al., 2016). While intravenous use of adrenalin more or less replicates the effects of SNS activity, blocking the cholinergic receptors of the PASY with atropin was reported to induce an unexpected rise in insulin sensitivity in one study (Svensson et al., 2011). This is possibly explained by differential effects of nicotinic and muscarinic receptors on peripheral insulin sensitivity. By and large though, chronic over-activation of SNS and/or under-activation of PASY would in theory raise blood glucose levels and thereby possibly contribute to the development of type 2 diabetes and other metabolic disorders.

Studies employing biochemical measurements of catecholamines as markers of SNS activity in subjects with insulin resistance, obesity and the metabolic syndrome have been inconsistent (Kjeldsen et al., 1983; Troisi et al., 1991; Tuck et al., 1992; Young et al., 1992; Tataranni et al., 1997; Lee et al., 2001; De Pergola et al., 2008; Surwit et al., 2010; Dai et al., 2012; Reimann et al., 2017). Those using measurement of sympathetic reactivity as reflected by levels of catecholamines in serum or urine after provocation by mental stress, cold stress or food intake have found some correlation to obesity (Schwartz et al., 1987), future development of obesity (Flaa et al., 2008b) and insulin resistance (Flaa et al., 2008a).

By using microneurography, muscle sympathetic nerve activity (MSNA) can be measured as pulse synchronous nerve bursts in resting muscles and constitute a surrogate measure for sympathetic activity in the whole body (Parati and Esler, 2012). Some studies using this method have demonstrated increased SNS in subjects with the metabolic syndrome compared to those without (Huggett et al., 2004; Grassi et al., 2009).

Heart rate variability can be computed by analyzing long-term or short-term ECG monitoring and used as a marker of sympathetic and parasympathetic activity and to reflect the balance of the two. The PASY and the SNS activity influence the heart rate variability in partially differential frequency bands. PASY activity is affected by the respiratory cycle and is detectable up to 1 Hz while SNS activity is largely dependent on input from baroreceptors, mediating a slower response up to 0.15 Hz. Therefore, the high frequency (HF) band of 0,15–0,40 Hz corresponds well to PASY activity while the low frequency (LF) parameter of 0.04–0.15 Hz is influenced by both SNS and PASY activity (Malik et al., 1996). There are several other indices of HRV (Kleiger et al., 2005). The ratio of LF/HF has been used to represent balance between SNS and PASY activity, although this has been criticized as being somewhat simplistic (Perini and Veicsteinas, 2003). Moreover, it has been pointed out that increased heart rate as such contributes to unfavorable HRV profiles, and that this must be taken into consideration when interpreting results from HRV studies (Monfredi et al., 2014). Henceforth, the term “low HRV,” although somewhat imprecise, will be used to describe an HRV profile indicative of relative over-activation of the SNS.

Several studies have shown a lower HRV in subjects with T2DM but whether HRV is a consequence of glycemic dysregulation or a causative mechanism behind the development of diabetes type 2 is not clear (Benichou et al., 2018). One study reported that reduced variability in the LF range was associated with increased risk of developing type 2 diabetes (Carnethon et al., 2003) while another study only found a correlation between baseline heart rate (and no other HRV indices) and the incidence of type 2 diabetes (Carnethon et al., 2006).

It is generally accepted that increasing insulin resistance precedes the development of overt type 2 diabetes. First degree relatives to patients with type 2 diabetes have a markedly increased risk of developing type 2 diabetes and are more insulin resistant than subjects with no family history of type 2 diabetes (Eriksson et al., 1989; Harrison et al., 2003). One study aimed at studying the association between insulin resistance and HRV in first-degree relatives of patients with diabetes type 2 compared with controls. While discovering no differences in insulin resistance in the groups, BMI and the LF/HF ratio were significantly and independently associated with insulin resistance (Svensson et al., 2016). Several other studies have found a correlation between measures of insulin resistance, glycemic status and HRV (Reims et al., 2004; Perciaccante et al., 2006; Stein et al., 2007; Thiyagarajan et al., 2012; Charles et al., 2013; Jarczok et al., 2013; Li et al., 2013; Cherkas et al., 2015; Indumathy et al., 2015; Meyer et al., 2016). In other studies, no correlation has been observed (Rannelli et al., 2017) or a differential correlation between men and women has been reported (Flanagan et al., 1999; Rannelli et al., 2017).

Multiple studies have also found an association between obesity and impaired HRV. BMI in itself has been significantly related to HRV in some studies (Molfino et al., 2009; Alrefaie, 2014; Hillebrand et al., 2015; Indumathy et al., 2015; Johncy et al., 2015) but not in others (Antelmi et al., 2004). Some studies suggest a stronger correlation between HRV and other anthropometric indices such as visceral adipose tissue, waist-hip ratio, waist circumference and body fat percentage: arguably markers that better reflect metabolically detrimental adiposity (Lindmark et al., 2005; Kimura et al., 2006; Sztajzel et al., 2009; Poliakova et al., 2012; Windham et al., 2012; Yi et al., 2013; Chintala et al., 2015; Indumathy et al., 2015; Yoo et al., 2016; Rastović et al., 2017; Yadav et al., 2017). Several studies have shown an improvement of HRV following weight-loss after surgery or by caloric restriction (Karason et al., 1999; Akehi et al., 2001; Ito et al., 2001; Nault et al., 2007; de Jonge et al., 2010; Sjoberg et al., 2011; Lips et al., 2013; Maser et al., 2013; Pontiroli et al., 2013; Casellini et al., 2016).

In a systematic review from 2014 on the correlation between HRV and the metabolic syndrome, the authors concluded that heart rate variability was generally reduced in females with the metabolic syndrome compared to those without while results in men were inconsistent (Stuckey et al., 2014). Since then, some studies have demonstrated a correlation between the metabolic syndrome and indices of low HRV (Ma et al., 2017; Carvalho et al., 2018) and one study has supported the differential correlations with regards to sex mentioned above (Stuckey et al., 2015). However, some studies suggest that the link between the metabolic syndrome and autonomic dysregulation as measured by HRV is fully explained by insulin resistance (Balcioğlu et al., 2016; Saito et al., 2017) and while low HRV was reported to predict the development of the metabolic syndrome in one study (Wulsin et al., 2016), another study demonstrated that this correlation was only significant for development of hyperglycemia and high blood pressure, whereas the risk of developing obesity and dyslipidemia were not significantly correlated to low HRV (Wulsin et al., 2015).

There is some support of ANS disturbances in various CNS disorders. Low employment status and high psychosocial stress have been associated with low HRV (Hemingway et al., 2005; Brosschot et al., 2007). An impaired HRV has also been reported in anxiety disorder (Chalmers et al., 2014), bipolar disorder (Faurholt-Jepsen et al., 2017), depression (Kemp et al., 2010), schizophrenia (Montaquila et al., 2015), and dementia (da Silva et al., 2018) although it has been suggested that this correlation may be confounded by the effects of medication (Sgoifo et al., 2015). Evidence of ANS and HPA dysregulation in CNS and metabolic disorders are summarized in Table 1.

**Table 1:** Summary of documented disturbances of the autonomic nervous system and the hypothalamus-pituitary-adrenal axis associated with CNS and metabolic disorders.

Disorder/condition	ANS disturbances	HPA disturbances	References
Psychosocial stress	↓LF, ↓HF associated with low employment grade; ↓RMSSD associated with psychosocial stress	Inconsistent findings	Hemingway et al., 2005; Brosschot et al., 2007; Joseph and Golden, 2017
Depression	↓HF, ↑LF/HF	↑Cortisol, ACTH; Flatted or blunted diurnal cortisol curve; ↑Cortisol awakening response in youths and adolescents; Improvement after treatment with SSRI; ↓ Cortisol reactivity	Kemp et al., 2010; Stetler and Miller, 2011; Joseph and Golden, 2017; Zorn et al., 2017
Anxiety disorder	↓HF	↓ Cortisol reactivity in females with anxiety disorder; ↑Cortisol reactivity in males with social anxiety disorder	Chalmers et al., 2014; Zorn et al., 2017
Bipolar disorder	↓LF	↑Cortisol and ↑ACTH, basal and after dexamethasone test	Belvederi Murri et al., 2016; Faurholt-Jepsen et al., 2017
Schizophrenia	↓HF; several studies in favor of HRV profiles indicative of normal SNS but reduced PASY	↓ Cortisol reactivity	Montaquila et al., 2015; Zorn et al., 2017
Dementia	↓RMSSD	↑Cortisol in Alzheimer’s	Notarianni, 2017;
Type 2 diabetes	↓RMSSD, ↓LF, ↓HF	Flatter diurnal cortisol curve; ↓Cortisol awakening response (Joseph and Golden, 2017)	Joseph and Golden, 2017; Benichou et al., 2018; da Silva et al., 2018
Insulin resistance	Cold pressor test and mental stress test predicative of future IR; ↓RMSSD; ↓HF;↑LF/HF; ↓↑LF	–	Reims et al., 2004; Perciaccante et al., 2006; Flaa et al., 2008a; Thiyagarajan et al., 2012; Charles et al., 2013; Jarczok et al., 2013; Li et al., 2013; Indumathy et al., 2015; Meyer et al., 2016; Svensson et al., 2016
Obesity	↓HF; ↑LF; ↓LF ↑LF/HF; ↓RMSSD	↑Cortisol reactivity; ↑Expression of 11βHSD1 ↑Hair cortisol levels	Molfino et al., 2009; Alrefaie, 2014; Wester et al., 2014; Hillebrand et al., 2015; Incollingo Rodriguez et al., 2015; Indumathy et al., 2015; Johncy et al., 2015; Jackson et al., 2017
Metabolic syndrome	↓LF; ↓HF; ↑LF/HF (females);↓RMSSD	↓Urinary free cortisol levels (females); ↑Hair cortisol levels	Abraham et al., 2013; Stuckey et al., 2014, 2015; Kuehl et al., 2015; Ma et al., 2017; Carvalho et al., 2018

*Only major results presented for reasons of clarity. HF, power in the high frequency range of HRV (>0,15 Hz), mostly affected by the parasympathetic nervous system (PASY); LF, power in the low frequency range (0,04–0,15), affected by activity in both PASY and the sympathetic nervous system (SNS); LF/HF, reflects the balance between SNS and PASY, high values reflecting relative SNS over-activity, due to either low PASY or high SNS activity; RMSSD, Root mean square of successive RR interval differences, mostly affected by PASY.*

The well-documented propensity of beta-blockers to cause insulin resistance and impair glycemic control in patients with diabetes (Lithell, 1991) and the fact that physical exercise improves insulin sensitivity in spite of increased SNS activity (Motahari-Tabari et al., 2014; Fisher et al., 2015) attest to the complicated relation between ANS disturbances and metabolic disorders. A troublesome aspect of studies utilizing measurement of HRV as methods is the plethora of indices available and the inconsistent manner in which these are used in different studies. This complicates comparison and somewhat limits the validity of the combined results.

**Table 2:** Summary of some important studies on CNS effects of nutrients and hormones on systemic glucose metabolism in humans.

Nutrient/hormone	Major findings	References
Glucose	↓ARC activity on fMRI directly after glucose ingestion predicted subsequent insulin levels; Hypoglycemia induced changes in hypothalamus activity prior to rise in CRH in healthy subjects; ↓Brain responses to hypoglycemia associated with attenuated levels of CRH in T1D with HU; CNS glucose uptake correlates negatively with insulin sensitivity	Dunn et al., 2007; Page et al., 2009; Osada et al., 2017; Boersma et al., 2018; Hwang et al., 2018
Insulin	Intranasal insulin leads to ↑insulin sensitivity and ↓HGP associated with changes in hypothalamic activity on fMRI and ↑PASY outflow; different CNS response to intranasal insulin and hyperinsulinemia in obese vs. lean subjects	Tschritter et al., 2006; Stingl et al., 2010; Heni et al., 2012, 2014b, 2017
GLP-1	Extra-pancreatic ↓HGP; GLP-1 receptors found in the CNS; fMRI-signs of altered CNS activity after administration	Prigeon et al., 2003; Alvarez et al., 2005; De Silva et al., 2011; van Bloemendaal et al., 2015; Farr et al., 2016a,b; Ten Kulve et al., 2016; Coveleskie et al., 2017

*ARC, arcuate nucleus; fMRI, functional magnetic resonance imaging; CRH, counter-regulatory hormones; T1D, type 1 diabetes; HU, hypoglycemic unawareness; HGP, hepatic glucose production; PASY, parasympathetic nervous system.*

### Disturbances of the Hypothalamus-Pituitary-Adrenal Axis in CNS and Metabolic Disorders

An excess of glucocorticoids, such as seen in Cushing’s disease or pharmacological glucocorticoid treatment, typically leads to insulin resistance and obesity as well as other features of the metabolic syndrome (Liu et al., 2014; Pivonello et al., 2016). This raises the question if elevated levels of glucocorticoids are also a feature in the “normal” pathophysiology of metabolic diseases. Glucocorticoids are produced and secreted by the adrenal cortex. This is regulated by the hypothalamus-pituitary-adrenal (HPA) axis in which hypothalamic release of CRH stimulates pituitary release of ACTH. This in turn stimulates glucocorticoid production and secretion. Feedback mechanisms occur at several levels. In the periphery, particularly in the liver and in adipose tissue, 11βHSD1 converts inactive cortisone to active cortisol whereas 11βHSD2 inactivates cortisol in a reverse manner, collectively providing an additional regulatory mechanism of glucocorticoid activity.

Circulating glucocorticoids act in several ways to disrupt normal glucose homeostasis. Glucocorticoids may cause beta cell dysfunction by influencing the uptake and metabolism of glucose in beta cells and may also attenuate the effects of GLP-1. In the periphery, glucocorticoids counteract insulin action by inducing insulin resistance through reduction of insulin downstream signaling substrates as well as indirectly by increasing lipolysis and proteolysis. Glucocorticoids also increase hepatic glucose production by activating enzymes involved in gluconeogenesis. Moreover, glucocorticoids also cause obesity and consequently insulin resistance by promoting differentiation and proliferation of adipocytes. Since glucocorticoid receptors are more abundant on visceral adipocytes, this favors visceral obesity (Di Dalmazi et al., 2012). Finally, excess glucocorticoids may lead to general weight gain (Berthon et al., 2014), probably at least partially by stimulating appetite through actions in the CNS, as is supported by a recent study (Serfling et al., 2019).

There is a multitude of ways to assess activity in the HPA-axis. These include plasma levels of cortisol, salivary levels of cortisol, 24 h urinary excretion of cortisol (UFC) and hair cortisol concentration (HCC). Activity in the HPA axis is highly variable and influenced by a range of factors including circadian rhythmicity, exercise, stress, and food-intake. Therefore, not only absolute concentrations of but also the diurnal curve of levels and dynamic changes in cortisol levels, as measured by cortisol reactivity to stress and dexamethasone suppression test, are interesting markers.

A few reviews on the topic have been published (Abraham et al., 2013; Incollingo Rodriguez et al., 2015) and will not be covered in detail here. From these and from original studies we can conclude that connections between obesity or the metabolic syndrome and basal levels of cortisol as measured in plasma, saliva, or urine are either inconsistent or disparate with regards to sex (Abraham et al., 2013; Incollingo Rodriguez et al., 2015). HCC is arguably a more reliable indicator of long-term levels of cortisol (Russell et al., 2015), in analogy with HbA1c, and studies using this outcome are more consistently in favor of such connections (Wester et al., 2014; Kuehl et al., 2015; Jackson et al., 2017). Moreover, consistently elevated cortisol reactivity tests in obese subjects as well as tendencies toward a flatter cortisol curve during day-time in obese subjects and toward impaired dexamethasone suppression tests in subjects with abdominal obesity was reported in one systematic review (Incollingo Rodriguez et al., 2015). Studies of HPA dysregulation in CNS disorder show variable results that are summarized in Table 1 (Stetler and Miller, 2011; Belvederi Murri et al., 2016; Joseph and Golden, 2017; Notarianni, 2017; Zorn et al., 2017).

In summary, long-term elevations, disturbances in the diurnal pattern, abnormal reactivity to stress and impaired feedback regulation of cortisol may have a small but limited impact on the development of obesity and metabolic disorder. Finally, there is substantial evidence that the expression of 11βHSD1 is upregulated in the adipose tissue of obese humans and animals. Thus, local dysregulation of glucocorticoids just as HPA dysregulation may contribute to the development of obesity and the metabolic syndrome (Incollingo Rodriguez et al., 2015).

**BOX 1 |** Neuroimaging techniques.

**Magnetoencephalography (MEG)**

This technique measures magnetic fields produced by electrical currents in the brain just as EEG measures electric fields. It is limited by the fact that cortical but not subcortical regions (including the hypothalamus) can be evaluated and has been largely replaced by other techniques to study the impact of metabolic input on brain activity.

**Functional Magnetic Resonance Imaging (fMRI)**

Blood oxygen level dependent (BOLD) signal is based on the fact that deoxygenated hemoglobin is paramagnetic whereas oxygenated hemoglobin is not, resulting in a higher signal in T2 weighted MRIs in situations with increased blood flow. Cerebral blood flow can also be measured by arterial spin labeling (ASL) where the water of incoming blood in for instance carotid arteries are labeled magnetically which changes the T1 signaling of water in the region that is examined.

The resolution in time and space is better than PET. Neuronal activity is not measured directly but via cerebral blood flow as a surrogate marker. By measuring spontaneous fluctuations in resting state brain activity, functional connectivity can be assessed and utilized to divide the brain into small functional units.

**Positron Emission Tomography (PET)**

This method measures gamma-rays that are emitted by unstable isotopes. Molecules that are labeled with positron-emitting isotopes are injected into the circulation. Fludeoxyglucose (18F) or FDG is widely used as a measure of glucose-uptake into tissues including the brain. To allow for neuroanatomic correlation of the signals, the method is combined with either CT or MRI and thus the space resolution does not differ from fMRI. However, the half-life of FDG is 110 min and it takes considerable time to generate a strong enough signal for the analysis. Hence, the time resolution is markedly limited compared to other methods.

**Single-Photon Emission Computed Tomography (SPECT)**

Similar to PET, this technique utilizes radioactive tracer material, but these emit gamma rays directly instead of positrons, limiting the spatial resolution as compared to PET.

### Central Effects of Glucose and Insulin on Systemic Glucose Metabolism: What Have We Learned From Neuroimaging Studies of Humans?

The methods employed in animal research previously discussed in this review are for obvious reasons not directly transferable to studies involving human subjects. The development and refinement of neuroimaging techniques has, however, offered an indirect way to explore changes in brain activity induced by and associated with metabolic changes. The neuroimaging techniques at hand (described in Box 1) have been extensively used to study CNS control of feeding and appetite which has been reviewed comprehensively elsewhere (De Silva et al., 2012; Zanchi et al., 2017). While PET is still used to a limited degree, fMRI has largely replaced other techniques as the method of choice for investigative purposes. Studies may use imaging of the whole brain or, more commonly, specific regions of interest (ROI) such as the hypothalamus. The small size and the location of the hypothalamus makes it difficult to image. In early studies, the hypothalamus was divided by a mid-sagittal slice resulting in the upper anterior, upper posterior, lower anterior, and lower posterior hypothalamus (Matsuda et al., 1999; Smeets et al., 2005). This division is too rough to allow for differential analysis of individual nuclei. However, a recent study utilized areal parcellation of the hypothalamus with a resolution of 1.25 mm × 1.25 mm and resting-state functional connectivity to generate 10 focii corresponding to different nuclei based on a previous histological study, demonstrating that such analysis of individual nuclei is indeed feasible (Osada et al., 2017).

It is not within the scope of this review to fully discuss the findings from the numerous studies aimed at exploring functional changes on brain activity as related to appetite or feeding control. However, a brief summary is warranted given the intimate relationship between energy intake and glucose regulation. A decrease in neural activity following glucose and meal intake has been reported in the hypothalamus and elsewhere (Matsuda et al., 1999; Tataranni et al., 1999; Gautier et al., 2000; Liu et al., 2000; Smeets et al., 2005, 2007; Flanagan et al., 2012; Page et al., 2013) that differs in obesity and type 2 diabetes according to some studies (Matsuda et al., 1999; Gautier et al., 2000; Vidarsdottir et al., 2007). Moreover, studies employing task-based fMRI protocols have demonstrated activation of several CNS regions (in particular the orbitofrontal cortex, amygdala, hippocampus and insula) upon presenting the subject with visual food cues (LaBar et al., 2001; Killgore et al., 2003; St-Onge et al., 2005; Porubská et al., 2006; Führer et al., 2008) which was attenuated in the fed state in some studies (Baldo and Kelley, 2007; De Silva et al., 2011) and different in obesity or insulin resistance in others (Killgore and Yurgelun-Todd, 2005; Rothemund et al., 2007; Stoeckel et al., 2008; Martin et al., 2010; Wallner-Liebmann et al., 2010; Dimitropoulos et al., 2012; Heni et al., 2014a; Alsaadi and Van Vugt, 2015). Caloric restriction normalized the hypothalamic response to glucose challenge in patients with type 2 diabetes in one study (Teeuwisse et al., 2012). Finally, appetite and feeding patterns in the CNS have been examined with respect to pharmacological administration and circulating levels of implicated hormones (Zanchi et al., 2017).

Collectively it seems that there are relatively well-characterized brain responses that are triggered by ingested and circulating levels of nutrients and hormones. Whether these brain responses have an actual effect on peripheral glucose metabolism has not, however, been investigated to a similar extent. The existence of the cephalic phase of insulin secretion as well as the observation that visual food-cues leads to lower postprandial glucose levels without affecting food-intake, insulin levels or other neuroendocrine parameters (Brede et al., 2017) is suggestive of such a response, the exact effector mechanisms of which remains elusive.

In the previously discussed study by Osada et al. (2017), 12 healthy subjects, both male and female, underwent fMRIs on two separates days following ingestion of 75 g of glucose solution or an equal volume of water. The hypothalamus was the region of interest and the imaging was analyzed by area parcellation, enabling analysis of individual nuclei. Thereby, the authors could report that the decrease in ARC activity between 0 and 10 min after the glucose ingestion significantly predicted insulin levels at 10 min. Thus, a causative link between the two events was plausible, according to the authors.

Hypoglycemia, on the other hand, was shown to induce changes in hypothalamic activity prior to the rise in counter-regulatory hormones (glucagon, cortisol, adrenaline, or noradrenaline) in healthy subjects (Page et al., 2009). The effect of hypoglycemia on CNS activation has been intensively studied in the setting of type 1 diabetes with unawareness. These patients have been shown to have a blunted rise in cerebral blood flow in hypoglycemia (Dunn et al., 2007; Mangia et al., 2012; Hwang et al., 2018) associated with a corresponding attenuation of counter-regulatory hormonal response (Mangia et al., 2012; Hwang et al., 2018).

While the intracerebroventricular administration of nutrients and hormones performed in animal studies is not an option in studies of humans, intranasal treatment of insulin preferentially targets the CNS, and thus provides an alternative for studying CNS effects isolated from systemic effects (Born et al., 2002). Intriguingly, intranasal insulin treatment has been demonstrated to lead to weight loss (Hallschmid et al., 2004), suppressed lipolysis (Iwen et al., 2014), positive cognitive results (Benedict et al., 2004; Hallschmid et al., 2008) and rapid reduction of glucose levels without any impact on C-peptide or insulin levels (Hallschmid et al., 2012). Heni et al. (2012, 2014b, 2017) have investigated the mechanisms behind this in a series of experiments where they showed an improvement of insulin sensitivity that was correlated with changes in hypothalamic blood flow on fMRI. An increase of HRV in the HF band was seen after intranasal insulin in one study, suggestive of augmented PASY outflow activity as a possible mediator between the CNS and the periphery (Heni et al., 2014b). In conflict with these studies, Ott et al. (2015) found no central effect of intranasal insulin aspart that could be discerned from its spillover effect in the periphery. Heni et al. (2018) recently reported yet unpublished results showing increased C-peptide levels 10 min after intranasal insulin administration during a hyperglycemic clamp in subjects with hypothalamic insulin sensitivity (as evidenced by a decrease of hypothalamic blood flow following administration) compared to those without. No reduction of C-peptide levels occurred in either group after administration, speaking against a substantial spill-over effect of intranasal insulin in this experiment (Heni et al., 2018).

Dash et al. (2015a) reported an inhibition of HPG 180 min after treatment with intranasal insulin, much later than what was seen in the studies by Heni et al. (2012, 2014b, 2017, 2018) According to the authors, this delayed response could be explained by a CNS-mediated effect on gene expression of proteins involved in hepatic gluconeogenesis rather than a more direct effect (Dash et al., 2015b). Similar kinetics of central insulin effects have been reported in dogs (Ramnanan et al., 2011) and rodents (Obici et al., 2002c). Moreover, the potent K_ATP_ channel opener diazoxide induced a 30% reduction of HGP after 6 h in humans in the settings of a pancreatic clamp, excluding the influence of pancreatic hormones and suggesting extra-pancreatic effects (Kishore et al., 2011). As previously discussed, intracellular insulin signaling in the hypothalamus is dependent on functional K_ATP_-channels and the brain may therefore be the extra-pancreatic site where diazoxide exerts its antidiabetic effects. In rats, ICV administration of diazoxide did indeed cause a reduction of HGP explained by downregulation of genes involved in gluconeogenesis (Kishore et al., 2011). Finally, human subjects with type 2 diabetes compared to controls did not exhibit suppression of HGP following diazoxide treatment in another study (Esterson et al., 2016), perhaps because of resistance to its extra-pancreatic effects.

There are several other findings suggesting the existence of CNS insulin resistance. A different response to intranasal insulin in obese vs. lean subjects has been reported in many studies (Hallschmid et al., 2004; Tschritter et al., 2006; Stingl et al., 2010; Heni et al., 2014b, 2017). In a recent study by our research team, whole-body ^18^FDG-PET was performed on patients with varying degrees of insulin sensitivity during a hyperinsulinemic-euglycemic clamp. Interestingly the *M*-value, an index of insulin sensitivity, correlated positively with uptake in muscles, adipose tissue and the liver and correlated negatively with glucose uptake in the brain (Boersma et al., 2018). This is corroborated by yet unpublished results by Rebelos et al. (2018). In their study of ^18^FDG-PET examinations in 151 subjects, the glucose uptake in the brain was correlated positively with BMI and negatively with insulin sensitivity. Of further interest, glucose uptake in the brain correlated positively with MRI signs of inflammation in the hypothalamus and amygdala (Rebelos et al., 2018), which is in consistency with another study (Thaler et al., 2012). Thus, hypothalamic inflammation may exist in obese humans in accordance with previously discussed findings in animal studies and may cause hypothalamic insulin resistance by the same proposed cellular mechanisms. Finally, Hirvonen et al. (2011) reported that brain glucose metabolism as assessed by ^18^FDG-PET increased in subjects with impaired glucose tolerance during a hyperinsulinemic-euglycemic clamp but not in healthy subjects, in whom the effect of insulin on brain glucose metabolism appeared to be saturated at fasting levels of insulin. Although this may suggest that insulin resistant subjects need more insulin to have a maximal effect on brain glucose metabolism, the fact that there was no difference in brain glucose metabolism at baseline between the two groups argues against the concept of such an insulin resistance in the CNS as a feature of systemic insulin resistance (Hirvonen et al., 2011).

Aside from the potential role of hypothalamic inflammation, the observation that the CSF:plasma ratio of insulin is inversely related to BMI (Kern et al., 2006) supports deficient transport of insulin as a cause for the putative hypothalamic insulin resistance. Table 2 summarizes some important studies of CNS effects of nutrients and hormones on systemic glucose metabolism in humans.

### Central Effects of Other Hormones, Bariatric Surgery and Pharmacological Agents on Systemic Glucose Metabolism

Leptin seems to regulate energy homeostasis in humans by central mechanisms (Rosenbaum et al., 2008; Grosshans et al., 2012) and to have an impact on whole-body glucose regulation in humans, as witnessed by its antidiabetic effects on patients with congenital leptin deficiency, congenital lipodystrophy, HIV-associated lipodystrophy and uncontrolled diabetes type 1 (Moon et al., 2013; Ramos-Lobo and Donato, 2017). However, it is not clear to which extent glucose regulation is carried out by peripheral vs. central mechanisms and no large effects have been demonstrated on the ANS (Eikelis et al., 2003; Machleidt et al., 2013) or the HPA-axis (Licinio et al., 1997; Sienkiewicz et al., 2011; Khan et al., 2012) that would grant indirect support to a contribution of central mechanisms. Oxytocin has demonstrated positive effects on various indices of glucose metabolism in several studies when given as a nasal spray (Ott et al., 2013; Lawson et al., 2015; Thienel et al., 2016), whereas the evidence from intravenous use is inconsistent (Lawson, 2017). Whether this is due to different central vs. peripheral effects on glucose metabolism and the preferential targeting of CNS by intranasal administration has not been illuminated.

The dopaminergic system has also been suggested to influence glucose homeostasis. The dopamine agonist bromocriptine-QR has been associated with an absolute reduction of HbA1c of 0,69% and is approved by the FDA for treatment of type 2 diabetes in adults (Garber et al., 2013). There are several possible mechanisms, probably working in concert. Firstly, dopamine receptors (D2R) are expressed in beta cells of the pancreas and activation of these have been shown to inhibit insulin secretion and cause an anti-proliferative effect (Rubi et al., 2005; Ustione et al., 2013). How this would lead to an improvement of glycemic indices is, however, challenging to comprehend. Secondly, activation of D2R in the pituitary gland reduces secretion of prolactin (excess of which is associated with hyperphagia and lipid accumulation in adipose tissue) and growth hormone (Woodside, 2007; Ben-Jonathan and Hugo, 2015). Thirdly, D2R-receptors are ubiquitous in the CNS where dopamine agonists have been shown to suppress levels of noradrenalin and serotonin in the hypothalamus (Luo et al., 1998), possibly resulting in reduced outflowing activity of the SNS from the brainstem to the periphery down the line. The dopaminergic system and D2R is also implicated in reward and appetite regulation (Baik, 2013). Interestingly, impairment of the dopaminergic system is a feature of Parkinson’s disease as well as an effect of antipsychotic medication and may partly explain the association of metabolic disorders with these conditions (Lopez Vicchi et al., 2016).

The propensity of bariatric surgery to improve glycemic control in patients is well-recognized and may offer insights regarding the development and pathogenesis of type 2 diabetes. Superiority of surgery to pharmacological approaches has been consistently documented in a vast number of randomized controlled trials (Koliaki et al., 2017). According to guidelines endorsed by several international diabetes organizations, so-labeled metabolic surgery in the treatment of patients with type 2 diabetes and BMI > 30 is advocated when lifestyle and pharmacological approaches has proved insufficient (Rubino et al., 2017). While still a matter of some controversy, there is substantial evidence indicating that the improvement of glucose indices seen after surgery is not fully explained by weight loss alone. The existence of weight independent mechanisms is suggested by the observed rapid improvement of glucose metabolism, occurring only days after surgery (Pories et al., 1995). Moreover, rates of diabetes remission differ depending on surgical technique, those bypassing the foregut, such as Roux-en-Y gastric bypass and biliopancreatic diversion being more efficacious in this regard than those who simply restricts the volume of the ventricle, such as gastric banding and gastric sleeve gastrectomy (Kodama et al., 2018).

A number of neuroimaging studies have demonstrated a profound impact of bariatric surgery on structural and functional features of the CNS. Obesity is associated with radiological signs of brain volume reduction and two studies have demonstrated normalization of these signs after bariatric surgery (Tuulari et al., 2016; Zhang et al., 2016). The enhanced response in reward regions to visual food-cues before surgery (and consistently demonstrated in obesity) have been reported to resolve after surgery (Scholtz et al., 2014; Frank et al., 2016). Two studies have shown an increase in these responses post-surgery following administration of octreotide (Goldstone et al., 2016) (an inhibitor of pancreatic hormones) and a GLP-1 receptor antagonist (Ten Kulve et al., 2017), respectively, suggesting altered secretion of gut hormones as a mediator. The role of GLP-1 in mediating satiety via the CNS is further supported by other neuroimaging studies (De Silva et al., 2011; van Bloemendaal et al., 2015; Farr et al., 2016a,b; Ten Kulve et al., 2016; Coveleskie et al., 2017). GLP-1 receptors are expressed in the brainstem and hypothalamus of humans (Alvarez et al., 2005) and some animal studies suggest that the effects of GLP-1 on reduction of food intake are partly if not primarily mediated by CNS actions (Kanoski et al., 2011; Secher et al., 2014). An effect on glucose metabolism independent of food intake has also been observed in animals (Sandoval et al., 2008) and in humans where a suppression of HGP without any measurable impact on pancreatic hormone levels has been reported (Prigeon et al., 2003).

The postprandial secretion of GLP-1 is augmented after bariatric surgery (Hutch and Sandoval, 2017). This is also true for oxyntomodulin (Laferrère et al., 2010), another gut hormone that has demonstrably induced weight loss in humans and rodents by reducing food intake and increasing energy expenditure (Dakin et al., 2001; Cohen et al., 2003; Baggio et al., 2004; Wynne et al., 2005, 2006). It appears to have a dual mechanism, acting on both GLP-1 and glucagon receptors (GCR) (Pocai, 2012). In rodents, a superior effect on weight loss was observed compared to GLP-1RA treatment alone at similar antihyperglycemic effects (Kosinski et al., 2012) making combined targeting of GLP1R and GCR attractive targets for future treatment of obesity as well as type 2 diabetes. Indeed, the combined agonist SAR425899 was recently evaluated in a phase 2 trial, reporting a significant weight loss and HbA1c reduction compared to placebo (Tillner et al., 2019). The concept of targeting glucagon receptors in the treatment of type 2 diabetes might be challenging to accept at first. Hyperglycemia or attenuation of the hypoglycemic effects of GLP-1R action would be a warranted concern. However, the improvement of glycemic indices reported in the study implies that the GLP-1R mediated effects on glucose homeostasis overrides those mediated by GCR. Intriguingly, glucagon injected into the CNS of rodents unexpectedly lowers systemic glucose levels by inhibition of HGP (Mighiu et al., 2013; LaPierre et al., 2015). Furthermore, intravenous infusion of glucagon leads to a transient hyperglycemia in rats, and this transiency is abolished when simultaneously blocking hypothalamic glucagon signaling (Mighiu et al., 2013). Thus, glucagon may actually have delayed CNS-mediated hypoglycemic effects opposing the acute hyperglycemic peripheral effects. The net effect of this could furthermore contribute to rather than impede the observed glycemic improvement of SAR425899.

The dual GIP/GLP-1 receptor agonists LY3298176 have been developed and recently demonstrated superiority to single GLP-1 RA treatment in terms of HbA1c reduction and weight loss (Frias et al., 2018). While considerably less is known about the CNS effects of GIP compared to GLP-1 and glucagon, dual GIP/GLP-1 RA treatment has shown positive effects on animal models of Alzheimer’s disease and Parkinson’s disease (Hölscher, 2018).

Circulating bile acids also increases after bariatric surgery (Jahansouz et al., 2016) and appear to have positive metabolic effects on their own accord (Sinclair et al., 2018). Bile acids have been demonstrated to cross the blood–brain-barrier where the expression of one of its receptors GPBAR1 has been reported (Keitel et al., 2010). Binding to FXR, another receptor, leads to positive metabolic effects in several organs and increases expression of FGF 15/19 from the foregut (Pournaras et al., 2012). As described previously, members of the FGF family have been shown to exert long-lasting and pronounced antidiabetic effects that appears to be mediated largely by central mechanisms (Fu et al., 2004; Morton et al., 2013; Marcelin et al., 2014; Scarlett et al., 2016). The targeting of bile acid signaling and in particular FGF signaling thus constitute promising future strategies in the treatment of type 2 diabetes.

There are a handful of drugs approved for treatment of obesity that primarily target the CNS. Among these, both lorcaserin and topiramate-phentermine have shown positive effects on glycemic indices. Lorcaserin, a 5HT-2c agonist was found non-inferior to several glucose-lowering drugs in terms of HbA1c reduction (0.55% for lorcaserin) and proportion of patients reaching target HbA1c in a recent meta-analysis (Neff et al., 2017). The authors conclude that lorcaserin should be considered as a second line add-on treatment in patients with type 2 diabetes and BMI > 27. A recently published RCT reported a more moderate reduction of HbA1c of 0.33% for patients with type 2 diabetes compared to placebo (Bohula et al., 2018). The combination of the amphetamine-like drug phentermine and the antiepileptic drug topiramate approved for treatment of obesity has likewise demonstrated a reduction in HbA1c of 4.4 mmol/mol compared to placebo in one study (Garvey et al., 2014a) and reduced progression to diabetes in patients with prediabetes and the metabolic syndrome was reported in another study (Garvey et al., 2014b). Although the antidiabetic effects of these CNS acting antiobesity drugs are relatively small-scale and probably a consequence of weight loss, an independent effect on glucose metabolism cannot be excluded at this stage.

Recently, the growth differentiating factor 15 (GDF15) has emerged as a satiety signal and potential target for treating obesity and metabolic disorders. It is secreted by adipocytes and many other cell types in response to cell injury and inflammation (Tsai et al., 2016). GDF15 has been demonstrated to induce weight loss and reduce insulin resistance in mice and primates by binding to its receptor GFRAL, the expression of which has not been demonstrated anywhere but the AP and the NTS in the brainstem (Mullican et al., 2017). Peripheral administration of GDF15 in mice induced neuronal activity in the AP and NTS and the anorexic effects were reversed by ablation of these regions (Tsai et al., 2014). Thus, existing evidence from animal studies indicate the brainstem as the target organ for GDF15. In humans, increased circulating levels of GDF15 has been observed as soon as 1 week after bariatric surgery and levels correlate linearly with insulin sensitivity both before and after surgery (Kleinert et al., 2019). Additionally, mitochondrial stress in mouse skeletal muscle cells increases the expression of GDF15 (Chung et al., 2017) and increased circulating levels in humans after physical exercise has been reported (Kleinert et al., 2018). As previously discussed, IL-6 and IL-10 also increase after exercise in humans and have been shown to mediate positive metabolic effects via the CNS in animals (Pedersen and Febbraio, 2008; Ropelle et al., 2010).

Physical exercise has been associated with altered brain fMRI responses to food-cues, favoring low-calorie foods over high calorie-foods (Killgore et al., 2013; Crabtree et al., 2014). While other studies demonstrating changes in brain fMRI responses to exercise are abundant, these have primarily focused on brain regions and processes involved in memory and learning, that are implicated in the pathogenesis of neurodegenerative disorders and dementia. To our knowledge, there are no neuroimaging studies that address the possible link between functional brain alterations and the well-recognized positive metabolic effects associated with physical exercise.

## Conclusion

Taken together, evidence from animal and human studies demonstrates that the brain detects levels of circulating nutrients and hormones and consequently organizes an outward response that contributes to the regulation of whole-body glucose homeostasis. However, there are major knowledge gaps about the exact nature of this response and its relative importance compared to peripheral processes. As we have seen, animal studies have provided an anatomical map of CNS glucose regulation and have identified important neurons and neural circuits involved. Additionally, the CNS sensing of key nutrients and hormones has been characterized in detail and the intracellular signaling pathways have been outlined for most of them.

Studies of humans entail inherent methodological challenges compared to animal studies that may explain the inconsistent findings in some areas. There are some intriguing epidemiological relationships between CNS and metabolic disorders and dysregulation of the ANS or HPA axis have both been proposed as the mediators of this connection. Attempts to demonstrate this have yielded somewhat discordant findings, however.

The extra-pancreatic effects of diazoxide and GLP-1 may very well be extra-cerebral as well and the favorable effect of intranasal oxytocin on glucose metabolism is interesting but not necessarily secondary to actions in the CNS. The use of somatostatin and octreotide in some studies to inhibit pancreatic hormone secretion may possibly induce CNS effects on their own accord and may thereby affect the results. The use of intranasal preparations of insulin arguably has a preferential CNS effect but dose-dependent spillover into the systemic circulation has been demonstrated in some studies that would make interpretation of results precarious.

Neuroimaging techniques have a large potential for further exploration of the interplay between CNS and systemic glucose regulation and have been used more extensively for investigation of appetite and feeding control with special regards to obesity. In these studies, resting-state or task-based neuroimaging is usually performed before and after some sort of metabolic intervention (i.e., OGTT or intranasal insulin) and the imaging is then compared for differences before vs. after the intervention and in between different subjects (i.e., lean vs. obese). Arguably, studies aimed at investigating acute CNS effects on glucose regulation must adopt a more sophisticated study design. Ideally, neuroimaging would be performed before vs. after some sort of metabolic intervention with concomitant measurements of hormonal activity and nutrients. If a detectable change in the activity of a brain region were to occur after the metabolic intervention but before a change in hormonal activity and nutrient levels, a causative link between the two events could be argued, in congruency with the results from Page et al. (2009) and Osada et al. (2017). A prerequisite for such a study design is that the utilized neuroimaging modality has a small enough time resolution to allow for such delicate temporal analysis, a description that fits fMRI but not PET. A matter of additional complexity is that the temporal relation between the CNS response to metabolic interventions and the putative peripheral effects is largely unknown and may differ between different stimuli. For instance, the response to hypoglycemia seems to be fast, occurring within 60 min in one study (Page et al., 2009), whereas the CNS-mediated insulin effects are not evident until after 180 min according to some studies (Obici et al., 2002c; Ramnanan et al., 2011; Dash et al., 2015a). Furthermore, the CNS response to shifts in nutrient availability and hormones may be conveyed to target organs by routes other than the ANS and the HPA-axis and alternative hormonal or neuronal pathways should at least be considered and evaluated in the future.

Other than in the work by Osada et al. (2017), the spatial resolution of fMRI protocols that were utilized in the reviewed studies have not permitted image analysis of separate nuclei of the hypothalamus but have either measured blood flow in the hypothalamus as a whole or in more crudely defined subdivisions of it. Since there is evidence that the relative proportion of GE and GI neurons varies in different nuclei (Mergenthaler et al., 2013), accurate subdivision of the hypothalamus may be imperative to produce reliable and relevant results. The mild inconsistencies between different neuroimaging studies is quite possibly a consequence of suboptimal resolution. This is best illustrated by the work of Heni et al. (2014b, 2017), in which one study examining the effect of intranasal insulin showed an increase of hypothalamic CBF whereas a decrease after administration was seen in another study. It is also important to underscore that the use of the surrogate markers CBF in fMRI and ^18^FDG uptake in PET may not correctly reflect changes in neuronal activity. The glucoCEST technique in MRI uses measurement of the proportion of phosphorylated and unphosphorylated 2-deoxy-D-glucose (2DG) and could thereby provide a better assessment of glucose metabolism than ^18^FDG-PET, while also embodying all the advantages with the fMRI modality. Likewise, MR spectroscopy provides a non-invasive method of measuring metabolite concentration in tissues and has for example been used to study hypothalamic glucose levels after hypoglycemic preconditioning (Seaquist et al., 2017). Nevertheless, neurons may exert either stimulatory or inhibitory downstream effects which are not discernable by methods limited to measurement of general neuronal activity, be that by proxy of cerebral blood flow or glucose metabolism.

In one study by Page et al. (2009), small reductions in systemic glucose levels induced increases in hypothalamic CBF before counter-regulatory hormones had risen. This raises the question: does this pattern differ in patients with glycemic dysregulation compared to normal subjects? The “brain-centric” model of development of type 2 diabetes stipulates that the hypothalamus regulates systemic glucose levels around a set-point that is skewed upwards as the disease progresses (Deem et al., 2017). Accordingly, glucose levels below this set-point would lead to processes aiming at raising glucose levels. Most likely, these processes would consist of the same counter-regulatory response that is mounted upon hypoglycemia in healthy individuals. It follows that the hypothalamic and counter-regulatory hormonal response would be activated at higher glucose levels in subjects with prediabetes compared to normal subjects. If this could be demonstrated in a hypoglycemic clamp during concomitant functional neuroimaging it would indeed lend strong support to the concepts of the “brain-centric” model. To our knowledge, this has not been demonstrated to date. Conversely, obese subjects exhibit different fMRI-responses to visual food cues during hyperglycemia than normal weight subjects according to one study (Belfort-DeAguiar et al., 2018). Whether the hypothalamic response to hyperglycemia differs in subjects with prediabetes compared to normal subjects and if such a possible difference corresponds to differences in hormonal activity remains to be investigated.

Since the brain is more or less dependent on glucose as a fuel substrate, its postulated ability to influence systemic glucose values or at least to correct hypoglycemia makes perfect sense from a teleological standpoint. As a consequence of the counter-regulatory response to hypoglycemia, systemic glucose levels are maintained at the expense of glucose uptake and utilization in insulin-sensitive organs such as muscles, adipose tissue and liver. Thus, the insulin-independent brain essentially prioritizes its own need for fuel over that of the rest of the body by mobilizing this counter-regulatory response. Expanding on this and on the “brain-centric” model, type 2 diabetes may be considered a condition in which the brain is “spoiled” by ever-increasing glucose levels and maintains those in a “selfish” fashion at the expense of the rest of the body.

In an elegant study published recently by Rodriguez-Diaz et al. (2018), pancreatic islets from three different species (humans, rhesus monkeys, and C57BL/6 mice) were transplanted to mice with streptozotocin-induced type 1 diabetes after which the glucose levels of the transplanted mice adapted to the normal ranges of the donors (varying considerably in between species). Thus, pancreatic islets were sufficient to regulate glucose levels on their own accord and this could not be overpowered by possible extra-pancreatic glucose regulation mechanisms. These findings indeed challenge the “brain-centric” model, but the transferability of these results from rodent models with experimental type 1 diabetes to humans with type 2 diabetes is a matter of reasonable doubt. Additionally, there may be brain-islet communication pathways that are species-specific and lost upon xenotransplantation.

Overall, there is robust evidence in favor of brain involvement in the regulation of glucose metabolism. Further investigations are needed to characterize the neurocircuits involved and the cross-talk with peripheral tissues. The use of refined neuroimaging methods is a promising way to test hypotheses derived from animal studies on human subjects. Such research will deepen our understanding of the pathogenesis of type 2 diabetes and potentially open up avenues for novel pharmacological approaches.

## Author Contributions

ML did most of the literature search. ML and JE conceived and wrote the manuscript. All authors contributed to literature search, reviewed and approved the final version.

## Conflict of Interest Statement

The authors declare that the research was conducted in the absence of any commercial or financial relationships that could be construed as a potential conflict of interest.
